# Therapeutic roles of thiazides and loop diuretics in blood pressure control and renal protection against chronic kidney disease

**DOI:** 10.1186/s40885-023-00238-5

**Published:** 2023-05-15

**Authors:** Wonji Jo, Eun Sil Koh, Sungjin Chung

**Affiliations:** Division of Nephrology, Department of Internal Medicine, Yeouido St. Mary’s Hospital, College of Medicine, The Catholic University of Korea, Seoul, Republic of Korea

**Keywords:** Hypertension, Chronic renal insufficiency, Kidney tubules, Diuretics, Antihypertensive agents

## Abstract

Fluid overload secondary to loss of functional nephron mass can elevate blood pressure, which is characteristic of hypertension shown in chronic kidney disease (CKD). Therefore, it is logical to use diuretics at appropriate dose to lower blood pressure in patients with CKD and hypertension. Despite the theoretical background on the use of diuretics in CKD, there have been no definitive data on the effectiveness or safety of diuretics as first-line therapy for the management of hypertension in patients with CKD. Results from some clinical trials have demonstrated that diuretics would not lower blood pressure. They could even worsen electrolyte imbalance and kidney function when they are administered in patients with CKD. Major clinical practice guidelines on management of blood pressure or CKD have stated that evidence for benefits of thiazide diuretics is not conclusive yet in patients with advanced CKD, although loop diuretics are often effective for volume control at lower glomerular filtration rate. Recently, evidence for diuretics as effective blood pressure lowering agents in patients with advanced CKD is increasing. Renoprotective effect of thiazide or loop diuretics might represent a consequence of their influence on blood pressure or their ability to potentiate the effect of renin-angiotensin system blockade by making intraglomerular pressure more renin-angiotensin system-dependent, although their direct benefit on renal function remains controversial. This review summarizes recent data on the possible role of diuretics in lowering blood pressure, slowing the progression of kidney disease, and reducing cardiovascular risk in CKD patients.

## Background

Hypertension is a well-known risk factor for chronic kidney disease (CKD) and cardiovascular disease [1, 2]. A recent study has confirmed that higher systolic and diastolic blood pressure are associated with an increased risk of CKD in a large population who have not taken any antihypertensive medication [3]. Therefore, early detection and proper management of hypertension are needed to reduce the risk of hypertension-related complications. Extracellular volume overload has been reported even in early stage of CKD [4]. However, a significant number of patients have subclinical volume overload without evident clinical signs [5, 6]. Such existence of extracellular volume increase can be detected by noticing impressive blood pressure reduction when diuretics are added in a situation of uncontrolled hypertension during the use of antihypertensive agents other than diuretics [4, 5]. Doubt on the effectiveness and safety related to use of diuretics in CKD patients has been present for many years. There have been insufficient data on the role of diuretics as first-line therapy for the treatment of high blood pressure in patients with CKD [7]. However, recent data have revealed that diuretics could be effective as antihypertensive agents in patients with advanced CKD [8, 9].

### Case 1

A 68-year-old woman with a long duration of type 2 diabetes and hypertension was referred to the Division of Nephrology with worsening renal function and proteinuria. She was taking azilsartan 80 mg, amlodipine 5 mg, rosuvastatin/ezetimibe 10/10 mg, nebivolol 5 mg, sitagliptin 50 mg, and extended-release metformin 1,000 mg with insulin injection. She had a blood pressure of 153/72 mmHg, a heart rate of 73 beats/min, and a respiratory rate of 16 breaths/min. Physical examination revealed regular heart beats without murmur, clear lung fields, and mild pitting edema. At presentation, she had the following results: serum creatinine, 1.61 mg/dL; estimated glomerular filtration (eGFR), 31.7 mL/min/1.73 m^2^; serum potassium level, 4.6 mmol/L; hemoglobin A1c, 7.1%; and 24-hour urine protein level, 5,481 mg/day. In addition to maintaining current medications, what drug should be provided to this patient?

Current evidence has shown that controlling blood pressure to be lower than the previous one could reduce cardiovascular events and all-cause mortality in patients with CKD [10–12]. According to Kidney Disease Improving Global Outcomes (KDIGO), adults with high blood pressure and CKD should be treated with a target systolic blood pressure < 120 mmHg using standardized office blood pressure measurement in the updated KDIGO 2021 Clinical Practice Guideline for the Management of Blood Pressure in Chronic Kidney Disease [7]. While previous guidelines have focused on the primary outcome of slowing CKD progression, nuance in blood pressure management of CKD patients recommended in the recent KDIGO guideline is the stronger emphasis on reduction of cardiovascular events and all-cause death rather than on renal protection. However, it is difficult to achieve the updated target for systolic blood pressure with only renin-angiotensin system (RAS) inhibitors and other available antihypertensive agents in practice, particularly in patients with more advanced CKD [13, 14], although current recommendations and general consensus on which medications to use for treating hypertension in CKD patients with or without diabetes and/or albuminuria have stressed the importance of initiating RAS inhibitors [7]. Therefore, the role of initial combination therapy in CKD patients appears to be clear since previous and recent guidelines have recommended that all patients with a blood pressure of 20/10 mmHg above the goal should be initiated with combinations of several antihypertensive drugs to enhance adherence and efficacy [15–17]. Along with much lower blood pressure target recommended by the recent KDIGO guideline, poorly controlled hypertension is becoming much more common than before in patients with CKD, especially for those with advanced stages. Physicians may face agonizing dilemmas on which antihypertensive combination is the best to prescribe. Unfortunately, there have been no randomized controlled trials comparing different drug combinations in CKD as there are no solid research studies on antihypertensive classes other than RAS inhibitors, β-blockers, and calcium channel blockers [7].

**Fig. 1:**
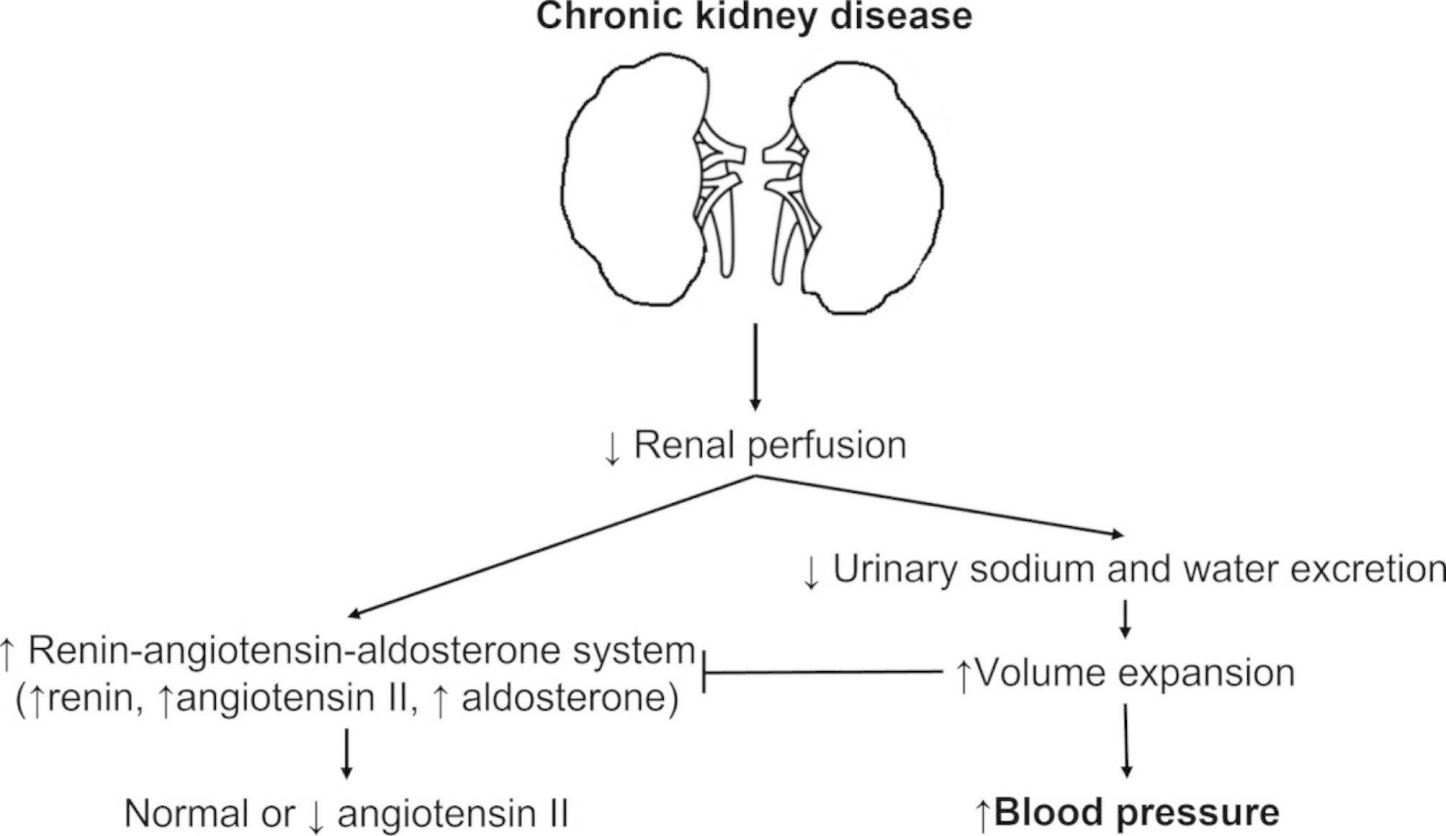
Schematic view of volume overload-induced hypertension in chronic kidney disease. Reduced glomerular filtration rate in chronic kidney disease eventually produces sodium retention and a fall in plasma renin level with minimal dependence of systemic angiotensin II, causing volume expansion and subsequent increased arterial pressure. In this situation, blockades of renin-angiotensin system are less effective in controlling systemic blood pressure

To find what combinations work the best for treating blood pressure in CKD, it is necessary to look again at the pathophysiology of hypertension in CKD. Numerous factors including genetics, pressure natriuresis, salt sensitivity, renin-angiotensin-aldosterone system, sympathetic nervous system, obesity, natriuretic peptides, endothelial dysfunction, arterial stiffness, and immune system have been thought to be mainly involved in the pathogenesis of hypertension [18]. Among them, a key factor in the regulation of blood pressure as a factor of cardiac output and systemic vascular resistance must be the phenomenon of pressure natriuresis, which is defined as an increase in sodium excretion from kidney because of mild increases in blood pressure, allowing blood pressure to remain in the normal range [19, 20]. For example, increased salt intake may cause an increase in extracellular volume and blood pressure. Subsequently, this increase in blood pressure will produce natriuresis, eventually restoring sodium balance and returning blood pressure to normal level. In some circumstances, this response may become abnormal whenever there is an abnormal sodium handling such as in conditions of reduced glomerular filtration rate (GFR), in which CKD is a representative example of this situation. In CKD, reduced perfusion of kidneys theoretically could cause sodium retention and subsequent activation of systemic RAS (Fig. 1). However, increased RAS would be offset by volume expansion resulting from decreased excretion of urinary sodium and water. As a result, systemic angiotensin II level in volume-expanded CKD might be rather normal or low. As CKD progresses, volume overload in whole body will likely worsen [21]. In this situation, it is natural that RAS blockades would be less effective for blood pressure control because of reduced systemic RAS. Rather, volume control could improve systemic blood pressure and sensitivity to RAS inhibitors in CKD patients with edema because RAS inhibitors would work only under a condition that systemic RAS is reactivated after volume depletion. As such, sodium retention, an inevitable consequence of reduced GFR, not only has a major role in the pathogenesis of uncontrolled hypertension in patients with CKD, but also precludes optimal control of blood pressure during pharmacological treatment with nondiuretic antihypertensive agents [22, 23]. Therefore, diuretics are logical agents at appropriate dosage to lower higher blood pressure in CKD [7].

## Antihypertensive effects of diuretics

**Fig. 2:**
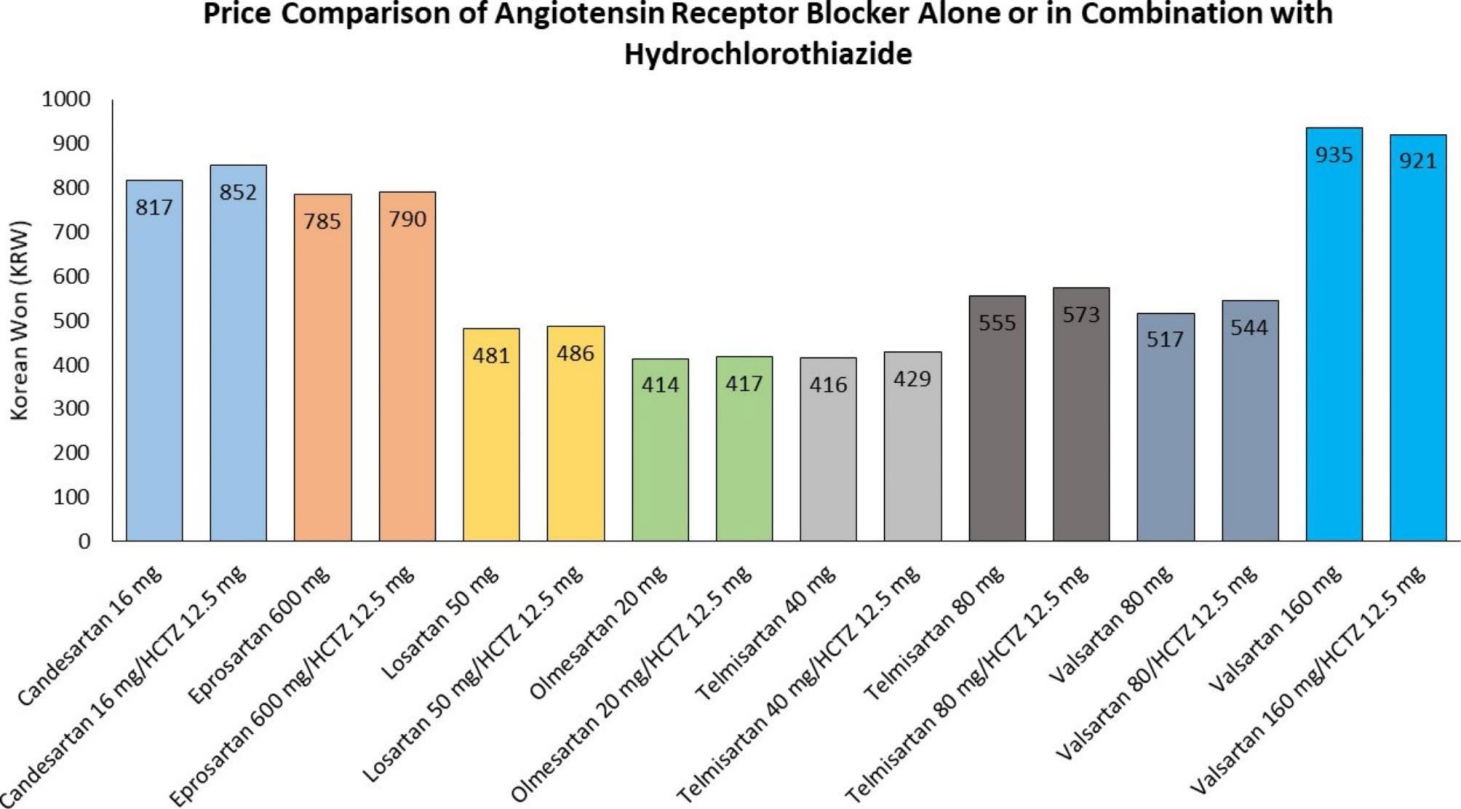
Price comparison of angiotensin receptor blocker alone or in fixed dose combination with hydrochlorothiazide (HCTZ) in Korea. Data on list prices of selected medicines were collected using a publicly available website provided by the Ministry of the Interior and Safety, Republic of Korea (https://www.data.go.kr/data/15067459/fileData.do). Brands for each agent were chosen based on the original drug developer that work in the Republic of Korea. The price of generic HCTZ 25 mg was KRW 10 in 2022. Price data were reported in 2022 KRW, and the KRW-US dollar rate was KRW 1,296/dollar in December 2022, based on the daily exchange rates provided by Woori Bank (https://spib.wooribank.com/pib/Dream?withyou=ENENG0358)

Oddly enough, diuretics for treating high blood pressure has been undervalued against other classes of antihypertensive agents for years. Although diuretics might have an advantage as an initial therapy for reducing cardiovascular events in certain racial or ethnic groups, there is a reluctance to reassess the “passé” drug in the era when RAS inhibitors such as angiotensin-converting enzyme inhibitor (ACEi) and angiotensin receptor blocker (ARB) are undoubtedly thought to be better than other agents [24–27]. When numerous clinical trials have evaluated the efficacy and safety of each antihypertensive agent, a diuretic agent has been mainly used for comparison. The Avoiding Cardiovascular Events Through Combination Therapy in Patients Living with Systolic Hypertension (ACCOMPLISH) trial comparing benazepril plus amlodipine group with benazepril plus hydrochlorothiazide has shown that the benazepril–amlodipine combination is superior to the benazepril–hydrochlorothiazide combination in reducing cardiovascular events or slowing CKD progression as well in patients with hypertension despite similar blood pressure control between groups [28, 29]. However, the adverse effect of diuretic on the progression of CKD or cardiovascular outcome has not been seen in all other studies. Post hoc analyses of a previous cardiovascular outcome trial have demonstrated that cardiovascular event rates are not higher in the diuretic group [30, 31]. The Antihypertensive and Lipid-Lowering Treatment to Prevent Heart Attack Trial (ALLHAT) has revealed that neither amlodipine nor lisinopril is superior to chlorthalidone in preventing major coronary events or in increasing survival [30]. In the ALLHAT, thiazide-type diuretics were proven to be unsurpassed in lowering blood pressure, reducing clinical events, and tolerability. Moreover, the cost-effectiveness appears to be another good thing about diuretics since single-pill combinations including a thiazide diuretic could cost no more than a nondiuretic component alone (Fig. 2).

## Antihypertensive effects of thiazide diuretics in chronic kidney disease

The term “thiazide diuretic” currently incorporates all diuretics believed to have a primary action on the Na-Cl cotransporter (NCC) in the distal tubule despite chemical structural variation among the heterogeneous group of agents including the thiazide-type diuretic hydrochlorothiazide as well as thiazide‐like diuretics such as metolazone and chlorthalidone [25, 32]. There has been a long-held belief that thiazide diuretics will lose efficacy in controlling diuresis and lowering blood pressure as GFR worsens [7]. Previous guidelines have recommended switching from thiazides to loop diuretics when GFR falls below 30 mL/min/1.73 m^2^ [33, 34]. However, it might be time to change the long-standing bias against the effectiveness of diuretics on blood pressure control in patients with CKD.

Although evidence against the use of thiazide diuretics in advanced CKD is still weak [7, 34], a few but significant trials have begun to test the role of thiazide diuretics in CKD (Table 1) [8, 35, 45, 50, 53, 55]. In a paper published in 2014, Agarwal et al. [35] from Indiana University investigated if chlorthalidone could result in improved 24-hour ambulatory blood pressure over 12 weeks among patients with moderate to severe CKD. Eligible subjects for their study were at least 18 years of age with eGFR between 20 and 45 mL/min/1.73 m^2^ and poorly controlled blood pressure, which was defined as systolic blood pressure ≥ 135 mmHg or diastolic blood pressure ≥ 85 mmHg by 24-hour ambulatory blood pressure monitoring despite taking ACEi or ARB and other classes of antihypertensive agents [35]. This pilot study has demonstrated that the 24-hour blood pressure in subjects with advanced CKD and resistant hypertension is significantly reduced by 10.5/3.1 mmHg after a 12-week treatment with chlorthalidone. The investigators also observed that albuminuria was significantly reduced by 40–45% with adverse events including electrolyte imbalance, transient creatinine change, and hyperglycemia. This pilot study helped design a subsequent, double-blind, randomized, placebo-controlled trial, the Chlorthalidone in Chronic Kidney Disease (CLICK) trial [8].

**Table 1:** Antihypertensive effects of thiazide and loop diuretics in subjects with CKD

Study	No. of subjects	Study duration (follow-up)	Renal function at baseline	Comparison	Outcome measure	Results
Thiazide diuretics (thiazide-type/thiazide-like)						
Agarwal et al. [35] (2014)	14	12 wk	eGFR 20–45 mL/min/1.73 m^2^	With vs. without chlorthalidone	24-hr ambulatory blood pressure	Decreased 10.5/3.1 mmHg with chlorthalidone
CLICK trial [8] (2021)	160	12 wk	eGFR 15–30 mL/min/1.73 m^2^	Placebo vs. chlorthalidone	24-hr ambulatory systolic blood pressure	Decreased 10.5 mmHg with chlorthalidone
Bennett et al. [45] (1977)	12	6 mo	Anuric kidney failure (CKD stage 5)	Hydrochlorothiazide or metolazone vs. placebo (crossover)	Predialysis and postdialysis blood pressure	No significant change in blood pressure
Loop diuretics						
Vasavada et al. [50] (2003)	14	9 wk	eGFR 42 ± 10 mL/min/1.73 m^2^ (CKD stage 2 to 3)	Torsemide vs. furosemide (crossover)	24-hr ambulatory blood pressure	No difference in blood pressure lowering
Dussol et al. [53] (2012)	23	3 mo	eGFR 25 ± 10 mL/min/1.73 m^2^ (CKD stage 3 to 4)	Furosemide vs. hydrochlorothiazide vs. combined regimen (crossover)	Mean blood pressure	No difference in blood pressure between two drugs (combined regimen has an additive effect)
Hayashi et al. [55] (2008)	19	Single or repeated doses	Anuric kidney failure (CKD stage 5)	Baseline vs. after furosemide administration	Systolic and diastolic blood pressure	No changes in blood pressure between before and after therapy

CKD, chronic kidney disease; eGFR, estimated glomerular filtration rate; CLICK, Chlorthalidone in Chronic Kidney Disease

In the CLICK trial, patients with stage 4 CKD defined as eGFR 15 to < 30 mL/min/1.73 m^2^ and uncontrolled hypertension as confirmed by 24-hour ambulatory blood pressure monitoring were included while receiving at least one antihypertensive drug [8]. The major finding of this trial was that the difference between the chlorthalidone group and the placebo group in the reduction of 24-hour systolic ambulatory blood pressure from baseline to 12 weeks was − 10.5 mmHg in favor of chlorthalidone group (Table 1). Most of the reduction in blood pressure occurred within 4 weeks after therapy using 12.5 mg of chlorthalidone was initiated. At 2 weeks after chlorthalidone therapy was discontinued, blood pressure remained below the baseline value. However, renal function returned to approximately the baseline value, suggesting additional involvement of tubuloglomerular feedback [8]. It was also observed that the reduction in the degree of albuminuria in the chlorthalidone group occurred within 4 weeks. Based on results of the CLICK trial, chlorthalidone must be an effective blood pressure lowering agent even in patients with advanced CKD. In the future, a larger trial of longer duration is needed to determine whether addition of chlorthalidone to a regimen of ACEi or ARB could further slow the progression of kidney disease and reduce cardiovascular risk without important safety concerns [9].

These small but influential results have made the recent KDIGO guideline to mention that several thiazide diuretics including chlorthalidone, metolazone, and indapamide appear to remain effective at GFR < 30 mL/min/1.73 m^2^ [7]. There are some differences in the volume of distribution and elimination half-life among thiazide diuretics. As a class, thiazides (including hydrochlorothiazide) and thiazide-like diuretics (including chlorthalidone, metolazone, and indapamide) have different chemical structures, which might be associated with their different characteristics. For example, chlorthalidone has longer duration of action and longer half-life elimination than hydrochlorothiazide [36]. It is expected to affect the extent and temporal pattern of blood pressure reduction, cardiovascular outcomes, or frequency of adverse events [37, 38]. Based on a few old studies, the 2017 American College of Cardiology/American Heart Association hypertension guideline has stated that chlorthalidone is preferred on the basis of longer half-life and proven trial reduction of cardiovascular disease [16]. However, the recent results of Diuretic Comparison Project (DCP) found that chlorthalidone use was not associated with major cardiovascular benefits when compared with hydrochlorothiazide [39]. On the other hand, chlorthalidone use was associated with greater risk of renal and electrolyte abnormalities [39, 40]. In addition, a population-based retrospective cohort study from Canada showed that chlorthalidone use was associated with a higher risk of eGFR decline, cardiovascular events, and hypokalemia compared with hydrochlorothiazide use [41]. Since there has been no clinical study targeting patients with CKD to examine whether all thiazide diuretics could have the same effect on blood pressure control and better clinical outcomes, the choice of the best one among thiazide diuretics for CKD is still unclear.

**Fig. 3:**
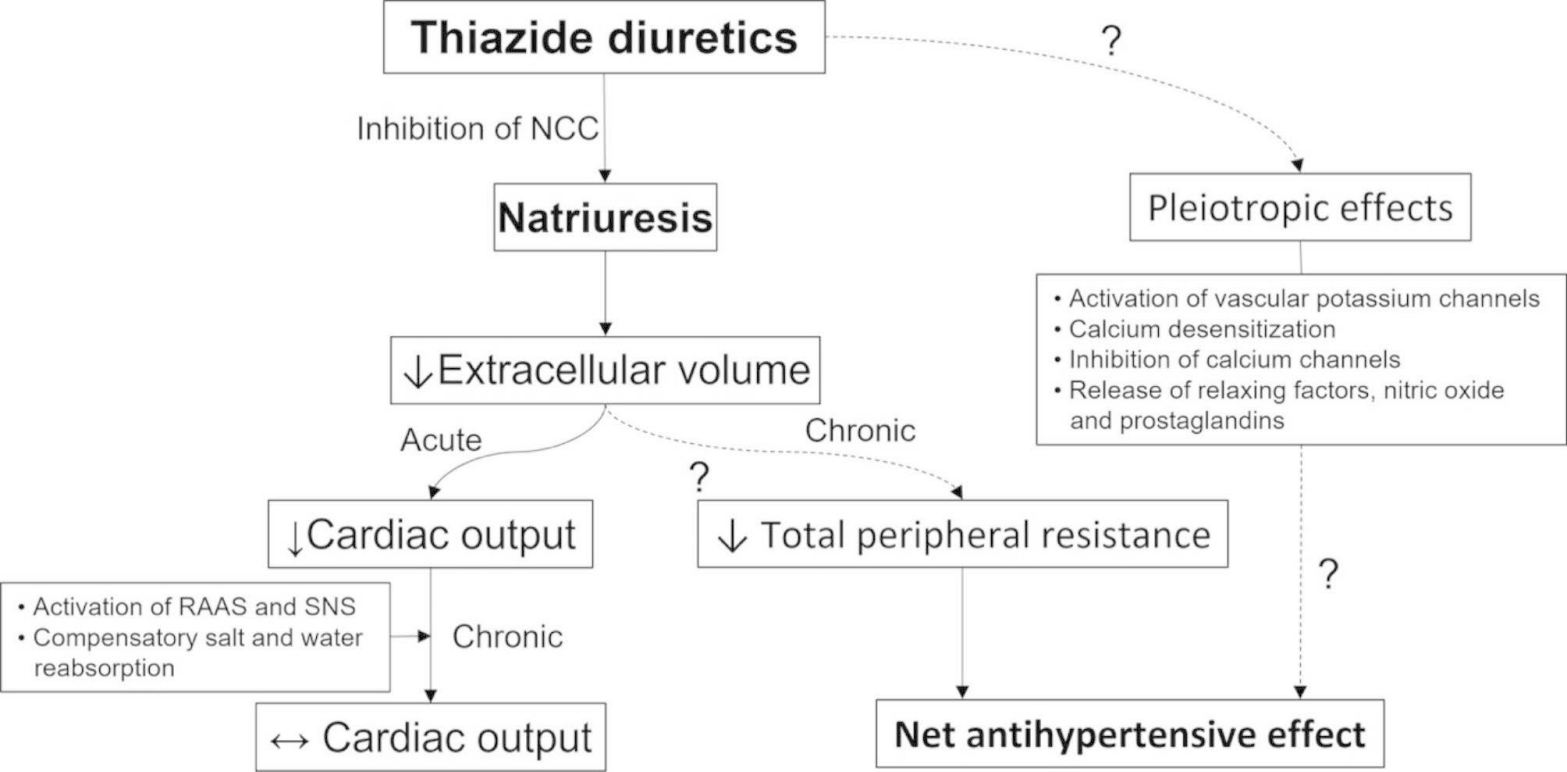
Proposed mechanisms responsible for blood pressure lowering effect with thiazide diuretics. After administration of thiazide diuretics, blood pressure is initially lowered due to a reduction in extracellular volume and subsequent cardiac output. However, within weeks, compensatory reabsorption of sodium and water can lead to return of the extracellular volume towards baseline. Antihypertensive effects of chronic thiazide therapy might be dependent of a fall of total peripheral resistance, a slight volume reduction and action on the vasculature. NCC, Na-Cl cotransporter; RAAS, renin-angiotensin-aldosterone system; SNS, sympathetic nervous system

Although thiazide diuretics have been proven to be effective even in patients with CKD than previously thought, the mechanisms responsible for the blood pressure lowering effect observed for thiazide diuretics are incompletely understood [37, 38]. After being rapidly absorbed by the gastrointestinal tract, thiazide diuretics are actively secreted through the renal organic anion transporter in renal proximal tubule [37]. Then they can inhibit sodium reabsorption by inhibiting NCC in the distal convoluted tubule, which is responsible for around 5% of total sodium reabsorption [38]. In renal failure, competition for anion transporter in the proximal tubule by accumulated organic anions could decrease the amount of thiazide diuretic that could reach the tubular fluid and then diminish its natriuretic effect [37]. Considering that chronic tubulointerstitial injury may exert more profound reduction on expression levels of numerous transporters and channels of the kidney as CKD progresses [42], the antihypertensive effect of thiazide diuretics does not appear to rely on inhibition of sodium reabsorption by blocking NCC. The mechanism for the ability of thiazide diuretics to acute lower of blood pressure is likely to be different from that of blood pressure lowering effect of a chronic therapy [37, 38, 43]. Thiazide diuretics can reduce blood pressure acutely by causing natriuresis, thereby reducing extracellular volume, venous return, and ultimately cardiac output [37, 38] (Fig. 3). In contrast, within 4 to 6 weeks of thiazide administration, compensatory salt and water reabsorption will return the extracellular volume towards baseline, which might be mediated by stimulation of the renin-angiotensin-aldosterone and sympathetic nervous systems resulting from thiazide-associated volume depletion [37]. The reason why the antihypertensive effect of thiazides persists even after normalization of the extracellular volume might be due to the fall in total peripheral resistance, which is caused by an unknown mechanism [38]. Vasodilatory effects such as activation of vascular potassium channels, opening of large conductance calcium-activated potassium channels, calcium desensitization, inhibition of voltage-dependent L-type calcium channels, release of endothelial-dependent relaxing factor and nitric oxide, and increased release of local vasodilatory factors such as prostaglandins have been suggested to contribute to enduring blood pressure lowering during chronic administration of thiazides [37, 38]. Older studies have also found that antihypertensive effects of thiazide diuretics are correlated with an increment of urinary kallikrein excretion rather than with volemic changes [44]. Nonetheless, given that daily administration of hydrochlorothiazide or metolazone to patients who are anuric on dialysis for 4 weeks could not improve blood pressure [45], natriuresis and subsequent slight reduction in volume rather than off-target effects would mainly contribute to persistent antihypertensive action of thiazides.

Going back to case 1 presented in the beginning of this paper, hydrochlorothiazide 12.5 mg was added to antihypertensive regimens of the patient. Three months later, she experienced a 9 mmHg decrease in her systolic blood pressure with a decrease in urine protein level down to 3,530 mg/day and an increase in serum creatinine level up to 1.91 mg/dL. It is unclear if the lower blood pressure by adding the diuretic could affect the long-term cardiovascular or renal outcome. However, if blood pressure is not controlled with initial antihypertensive agents, the addition of a thiazide diuretic would be a reasonable and effective step [38].

## Antihypertensive effects of loop diuretics in chronic kidney disease

Compared with thiazide diuretics, loop diuretics are known to have relatively short acting duration, limiting their widespread adoption for generally treating hypertension [7, 38]. As has previously been explained, a loop diuretic might be more useful for reducing volume overload and then decreasing blood pressure in conditions of extracellular volume expansion such as CKD. Loop diuretics can exert their effects by inhibiting the Na-K-2Cl cotransporter (NKCC2) in the apical membrane of the thick ascending limb of the loop of Henle in kidneys, which is known to be responsible for around 25% of renal sodium reabsorption under normal conditions [38]. Natriuresis and diuresis by a loop diuretic can lead to a decrease in effective circulating volume, causing a fall in cardiac output and an increase in pulmonary vascular resistance [46]. In fact, the overall effect of loop diuretics is complex and not entirely clear. Several factors including direct inhibition of NKCC2 after a loop diuretic administration can increase renin release. Conversely, the increase of lumina sodium concentration at the level of macula densa could reduce renin release, leaving uncertainty about the net effect of a loop diuretic on renin release [46]. In case of an increase of renin release, subsequent activation of angiotensin and aldosterone would be linked to arterial vasoconstriction, the opposite effect of arterial vasodilation [47, 48]. Similar to the mechanism of blood pressure lowering effect after chronic administration of thiazide diuretics, blood pressure lowering with chronic therapy of loop diuretics in CKD might involve both volume regulation and vascular effects [37]. Vascular responses by a loop diuretic may include both direct and indirect effects such as increased venous compliance, increased urate level, and decreased skin sodium [37, 46]. As these all seem to be able to have reciprocal effects on each, long-term effects of loop diuretics on sodium balance, extracellular fluid volume, and blood pressure become unpredictable [46]. This might explain why there are few clinical studies on blood pressure lowering efficacy of loop diuretics.

When using diuretics in CKD, it may be preferable to choose torsemide over furosemide because torsemide has a longer duration of action [38]. Torsemide has been reported to exhibit a bioavailability of 90–100% regardless of whether patients have CKD [49]. Furthermore, its large component of nonrenal clearance makes elimination half-life of torsemide unchanged in patients with CKD [50]. In contrast, the bioavailability of furosemide is more decreased in patients with CKD compared with that in patients with normal kidney function [50]. In addition, the elimination half-life of furosemide is prolonged as kidney function decreases [51]. However, a few comparative studies on natriuretic and blood pressure lowering effects between loop diuretics in patients with CKD have shown conflicting results [50, 52]. A previous randomized, double-blind, two-period, crossover trial has failed to show superiority of torsemide over furosemide with respect to natriuresis or 24-hour ambulatory blood pressure control in patients with stage 2 or 3 CKD [50]. On the other hand, a recent systemic review and meta-analysis including all published studies that compared torsemide and furosemide use in heart failure patients (although not targeting at patients with CKD) showed that torsemide use was associated with significantly more improvement in functional status and lower cardiac mortality in patients with heart failure compared with furosemide use [52]. In this analysis, patients who received torsemide among included patients were more likely to have CKD compared with patients who received furosemide (42.4% vs. 32.6%), displaying a current tendency to prescribe torsemide more frequently than furosemide.

Since the change in diuretic therapy from thiazides to loop diuretics when GFR declined below 30 ml/min/1.73 m^2^, a tradition at one time, turned out to be unnecessary as noted above, one might wonder which of thiazide diuretics and loop diuretics could be a better choice for treating hypertension in CKD. A randomized, double-blind, crossover trial has compared fractional excretion of sodium and chloride after chronic administration of furosemide and hydrochlorothiazide [53]. In that study, mean blood pressure decreased by the same extent after administration of furosemide and hydrochlorothiazide (from 101 mm Hg to 93 mm Hg and 94 mm Hg, respectively), showing that natriuretic and antihypertensive responses to each drug were similar. As expected, the combination of furosemide and hydrochlorothiazide had an additive effect on natriuresis and blood pressure [53]. These additive effect of the combined loop and thiazide diuretics on antihypertensive and diuretic actions could be vital, especially in the setting of refractory volume overload seen in advanced CKD, congestive heart failure, and end-stage liver disease [3].

In the case of “kidney failure”, a more appropriate term than previously used term of “end-stage renal disease” or “end-stage kidney disease” [54], loop diuretics have been continuously used in practice without solid evidences even after starting dialysis to help address volume overload when the residual urine output is still preserved [38]. As with thiazide diuretics, a previous study has demonstrated that neither low doses nor high doses of furosemide in patients with anuric kidney failure undergoing hemodialysis can induce any significant changes in systolic or diastolic blood pressure [55].

Outpatient visit-to-visit blood pressure variability (BPV) has been reported to be independently associated with poor cardiovascular outcomes in the general population [56, 57]. Although such data in CKD patients are scarce, advanced CKD patients treated with diuretics show lower BPV than those treated with drugs of other classes [58, 59]. In addition, a recent observational cohort study using real-world clinical data from a national sample of 62,788 US veterans with prevalent non–dialysis CKD stages 1 to 5 has verified that BPV is strongly associated with composite cardiovascular events, all-cause death, cardiovascular death, myocardial infarction, hospitalization for heart failure, and ischemic stroke, but not progression of CKD to kidney failure requiring kidney replacement therapy in patients with non–dialysis CKD [59]. The study found that thiazide or loop diuretic-based antihypertensive regimens were not associated with decreased BPV compared with nondiuretic regimens, although such regimens did modify the association of BPV with cardiovascular events at the highest BPV quintiles [59]. It is not yet absolutely clear what diuretic works better or what combination of antihypertensive regimens works better. Thus, more research is needed.

## Renoprotective properties of diuretics

### Case 2

A 64-year-old man with an unknown duration of diabetes visited a clinic with uncontrolled blood pressure, decreased visual acuity, and generalized edema. His blood pressure was 159/86 mmHg and his heart rate was 88 beats/min. He was noted to have decreased kidney function with a serum creatinine level of 3.70 mg/dL, an eGFR of 18.0 mL/min/1.73 m^2^, and a spot urine protein to creatinine ratio of 9,190 mg/g. Kidney biopsy showed typical diabetic nephropathy with 90% glomerular sclerosis, grade 3 of tubular atrophy, grade 3 of interstitial fibrosis, and grade 3 of fibrous wall thickening of vessels. ARB was started but immediately discontinued due to a fast increase in serum creatinine by more than 40%. He was put on cilnidipine, carvedilol, furosemide, and hydrochlorothiazide. On visit after 8 months, pitting edema of lower extremities was not observed. He had a blood pressure of 125/83 mmHg and a serum creatinine level of 4.07 mg/dL. His doctor was worried about if long-term use of diuretics could wreak havoc on this patient’s renal function and asked an expert opinion. How should you as an expert respond to this issue?

## Effects of thiazide and loop diuretics on proteinuria and renal function in chronic kidney disease

Since RAS inhibitors are superior to other classes of antihypertensive agents in patients with high blood pressure and CKD for kidney and cardiovascular outcomes with or without diabetes and albuminuria, renoprotective potentials of diuretics have had quite a low profile. The only diuretics that have been investigated in large clinical trials with hard end points for antiproteinuric and renoprotective effects have been mineralocorticoid receptor antagonists such as spironolactone, eplerenone, and finerenone [60]. However, other types of diuretics including thiazides have also shown significant antiproteinuric effects, although most studies have been performed in a short term. In a previous study including nondiabetic patients with proteinuria of more than 1 g/day and a creatinine clearance of more than 30 mL/min during chronic ACEi treatment, the addition of hydrochlorothiazide to high salt intake resulted in a reduction in blood pressure of 10% and proteinuria of almost 40% whereas the reduction in creatinine clearance was more than 20% (Table 2) [8, 35, 61–68]. Another study showed that the addition of thiazide diuretics to ACEi or ARB in patients with immunoglobulin A nephropathy restored nocturnal blood pressure decline and reduced proteinuria [62]. Since that study included only patients with preserved renal function, no difference in creatinine clearance between before and after adding diuretics was observed. Hydrochlorothiazide added to ARB showed an efficacy on par with low sodium diet in reducing blood pressure and proteinuria. Furthermore, the largest effect on proteinuria and blood pressure was obtained during their combination in proteinuric patients with stable renal function without diabetes [63]. Whatever sodium-depleting measures were, a fall in creatinine clearance was observed. In a double-blind, placebo-controlled, crossover randomized trial involving type 2 diabetic nephropathy patients with albuminuria and creatinine clearance of more than 30 mL/min, treatment with sodium restriction or hydrochlorothiazide significantly reduced albuminuria and their combination reduced albuminuria further than either treatment alone [64]. While renal function remained unaffected by sodium restriction or hydrochlorothiazide, their combination significantly reduced creatinine clearance. This decrease was reversible upon their discontinuation [56]. Since these were all short-term trials with patients whose renal function was relatively preserved, long-term effects of thiazides on proteinuria reduction and preservation of renal function were unclear. When effects of adding thiazides to antihypertensive medications including loop diuretics in type 2 diabetic patients with CKD stage 4 to 5 were examined, blood pressure and proteinuria as well as edema were all improved at 12 months after initiating hydrochlorothiazide [65]. Researchers of that study claimed that, although eGFR gradually decreased during the study, the annual eGFR decline was not significantly different between before and after hydrochlorothiazide initiation [65].

**Table 2:** Effects of thiazide and loop diuretics on proteinuria or albuminuria in subjects with CKD

Study	No. of subjects	Study duration (follow-up)	Renal function and albuminuria (proteinuria) at baseline	Comparison	Outcome measure	Results
Thiazide diuretics (thiazide-type/thiazide-like)						
Buter et al. [61] (1998)	7	12 wk	Creatinine clearance 51–101 mL/min with proteinuria of 1.4–5.6 g/day	Low sodium vs. high sodium vs. high sodium intake plus hydrochlorothiazide, with ACEi	Creatinine clearance, 24-hr proteinuria	Decreased mean 14 mL/min in creatinine clearance Decreased mean 1.7 g/day in 24-hr proteinuria after addition of hydrochlorothiazide to high sodium intake compared with high salt intake alone
Uzu et al. [62] (2005)	25	8 wk	Serum creatinine ≤ 1.2 mg/dL with proteinuria of 0.5–3.0 g/day	ACEi vs. ARB, with and without trichlormethiazide	Creatinine clearance, 24-hr proteinuria	No difference in creatinine clearance Decreased 0.48 g/day in proteinuria after addition of thiazide
Vogt et al. [63] (2008)	34	36 wk	Creatinine clearance > 30 mL/min with proteinuria of 2–10 g/day	Placebo vs. ARB vs. ARB plus hydrochlorothiazide, during high salt or low salt diet (crossover)	24-hr proteinuria, serum creatinine	Decreased proteinuria by 56% with adding hydrochlorothiazide and by 70% with combined addition of hydrochlorothiazide and low-sodium diet Same pattern in change of creatinine clearance after adding hydrochlorothiazide
Kwakernaak et al. [64] (2014)	45	30 wk	Creatinine clearance 101 ± 47 mL/min/1.73 m^2^ with mean albuminuria of 711 mg/day	Placebo vs. hydrochlorothiazide, with regular sodium or sodium restriction, during background ACEi (crossover)	Albuminuria, renal function	Decreased mean albuminuria to 393 mg/day by sodium restriction, 434 mg/day by hydrochlorothiazide and 306 mg/day by their combination Creatinine clearance unaffected by sodium restriction but decreased 14 mL/min by the combination
Hoshino et al. [65] (2015)	11	12 mo	eGFR 21.5 ± 8.1 mL/min/1.73 m^2^ with proteinuria 6.7 ± 3.9 g/g	Addition of hydrochlorothiazide to existing antihypertensive medication including loop diuretics	Proteinuria, eGFR	Decreased 4.3 g/g in proteinuria Decreased 8.4 mL/min/1.73 m^2^ in eGFR after initiation of hydrochlorothiazide
Agarwal et al. [35] (2014)	14	12 wk	eGFR 20–45 mL/min/1.73 m^2^ with mean baseline urine albumin excretion rate 604 mg/g during day and 535 mg/g during night	With vs. without chlorthalidone	Urine albumin to creatinine ratio	Decreased albuminuria by 40–45% Transient increase in plasma creatinine by 0.24 ± 0.14 mg/mL at week 8 (returning to baseline at week 12) in chlorthalidone group
CLICK trial [8] (2021)	160	12 wk	eGFR 23.2 ± 4.2 mL/min/1.73 m^2^ with urine albumin excretion rate 862 mg/g for chlorthalidone and 812 mg/g for placebo	Placebo vs. chlorthalidone	Urine albumin to creatinine ratio, eGFR	Decreased albuminuria by 52% in chlorthalidone group Between-group difference by − 2.2 mL/min/1.73 m^2^ in eGFR (lower in chlorthalidone group)
Marre et al. [66] (2004)	570	1 yr	Creatinine clearance 91.5 ± 29.5 mL/min and urine albumin to creatinine ratio 6.16 mg/mmol in indapamide group; 93.4 ± 29.2 mL/min and urine albumin to creatinine ratio 6.17 mg/mmol in ACEi group	Indapamide sustained release vs. enalapril	Urine albumin to creatinine ratio, creatinine clearance	Improvement to normoalbuminuria by 40% in indapamide group and by 42% in ACEi group without change in creatinine clearance between two groups
Loop diuretics						
Esnault et al. [67] (2005)	18	8 wk	Serum creatinine 151.22 ± 63.9 µmol/L with 24-hr proteinuria 3.71 ± 2.1 g/day	Ramipril 5 mg vs. ramipril 10 mg vs. valsartan 160 mg vs. combined of ramipril 5 mg and valsartan 80 mg vs. combined of ramipril 5 mg and valsartan 80 mg plus increased furosemide dosage (20–80 mg)	Urine protein to creatinine ratio, 24-hr proteinuria, serum creatinine	Decreased proteinuria by 18.9% in combined ramipril and valsartan group and by 44.5% in combined ramipril, valsartan and increased furosemide Increased serum creatinine by 7.6% in combined ramipril and valsartan group and by 26.2% in combined ramipril, valsartan and increased furosemide
Esnault et al. [68] (2010)	18	18 wk	eGFR 39.2 mL/min/1.73 m^2^ with 24-hr proteinuria 1.97 g/day	Combined ramipril 5 mg and valsartan 80 mg vs. combined ramipril 10 mg and valsartan 160 mg vs. combined ramipril 5 mg, valsartan 80 mg and increased furosemide dosage	Urine protein to creatinine ratio, 24-hr proteinuria, eGFR	Proteinuria 1.95 g/day by combining low doses of ramipril and valsartan vs. 1.75 g/day by combining higher doses ramipril and valsartan vs. 1.20 g/day by combining lower doses of ramipril and valsartan plus increased furosemide dosage eGFR 40.4 mL/min/1.73 m^2^ by combining lower doses of ramipril and valsartan vs. eGFR 38.1 mL/min/1.73 m^2^ by combining higher doses of ramipril and valsartan vs. eGFR 33.4 mL/min/1.73 m^2^ by combining lower doses of ramipril and valsartan plus increased furosemide dosage

CKD, chronic kidney disease; ACEi, angiotensin-converting enzyme inhibitor; ARB, angiotensin receptor blocker; eGFR, estimated glomerular filtration rate; CLICK: Chlorthalidone in Chronic Kidney Disease

Renoprotective effects of thiazide-like diuretics have also been reported. Based on data from the CLICK trial and its pilot trial targeting patients with advanced CKD, the administration of chlorthalidone could lead to a reduction in urine albumin excretion [8, 35]. Adverse events known to be associated with chlorthalidone therapy such as an increase in serum creatinine level occurred more frequently in the chlorthalidone group than in the placebo group [8, 35]. Interestingly, such a concomitant rise in serum creatinine at about the middle of the study might be attributed to volume depletion, followed by a gradual improvement and return to baseline at the end of the study, suggesting that renal deterioration after treatment with chlorthalidone could be reversible [35]. The subsequent CLICK trial also observed reversible changes in eGFR and reduction in the degree of albuminuria in the chlorthalidone group [8]. Due to short duration and relatively small size of these studies, care should be exercised when interpreting these data. When the Natrilix SR Versus Enalapril Study in Type 2 Diabetic Hypertensives with Microalbuminuria (NESTOR) study compared efficacies of two antihypertensive drugs, indapamide and ACEi, indapamide-based therapy was found to be equivalent to ACEi-based therapy in reducing microalbuminuria in type 2 diabetic patients with hypertension [66]. In that study, renal function did not change in either treatment group.

Unlike thiazide diuretics, loop diuretics have little evidence to support its antiproteinuric effects (Table 2). Increased furosemide dosage in addition to combined half doses of ACEi and ARB in patients with proteinuric CKD enabled a better control of proteinuria than uptitration to the full dose of ACEi and ARB [60, 67, 68]. This antiproteinuric effect by loop diuretics accompanied both decreases in blood pressure and eGFR [67, 68].

The precise mechanisms by which thiazide or loop diuretics have antiproteinuric effects have not been clarified yet. The effect of a sodium load that can inhibit the antiproteinuric effect of RAS blockades could be restored by diuretics [67]. In addition, the improvement in blood pressure response during thiazide diuretics might contribute to the reduction in proteinuria [61]. Based on data from the CLICK trial, the increase in albuminuria from the time chlorthalidone therapy was discontinued to 2 weeks later could suggest that the mechanism of the reduction in the degree of albuminuria was at least in part hemodynamically mediated [8]. Lowering of blood pressure by thiazide is likely evoked by lowering of extracellular fluid volume as shown by lowering of total body volume and B-type natriuretic peptide. Mitigation of these effects over time suggests nonvolume mechanisms such as lowering vascular resistance to maintain the blood pressure lowering effect [35]. However, considering that the addition of thiazides resulted in a reduction in blood pressure of 10% whereas the reduction in proteinuria was 40%, the antiproteinuric effect by thiazide diuretics could not be explained solely by a systemic blood pressure lowering effect [61]. The inevitable but reversible acute drop in eGFR along with the reduction in albuminuria by chlorthalidone therapy indicates that chlorthalidone might lower intraglomerular pressure in the same way as other classes of drugs such as ACEi, ARB, and sodium-glucose cotransporter inhibitors with proven renoprotective actions [9].

It also remains that the effect of loop diuretics on proteinuria is independent of its diuretic property. Like thiazide diuretics, chronic use loop diuretics in CKD might contribute to a reduction of proteinuria by lowering blood pressure with subsequent volume regulation [37, 69]. The beneficial effect of loop diuretics on proteinuria could also be partly explained by an eGFR decrease, leading to hemodynamic modifications [67, 68]. There has been no sharp evidence showing that thiazide and loop diuretics per se are capable of lowering proteinuria. It is well-known that sodium restriction is effective in increasing efficacy of ACEi or ARB [60, 64]. Thus, the mechanism of action of loop diuretics might involve their ability to potentiate the effect of RAS blockade by making intraglomerular pressure more RAS-dependent through their natriuretic and diuretic effects. Blood pressure might have also been reduced to the same extent by ACEi and diuretic having opposite effects on the RAS [66, 69]. To reduce proteinuria, effective reduction of blood pressure is still a matter of the greatest importance.

## Diuretics and risk of chronic kidney disease progression and kidney failure

One of the reasons why physicians hesitate to use diuretics acutely or chronically is because whether diuretics could result in direct kidney injury or just benign hemoconcentration of serum creatinine by volume depletion remains controversial [70]. Especially, one might wonder if chronic administration of diuretics in patients with CKD is associated with CKD progression and/or increased risk of kidney failure requiring kidney replacement therapy [70]. As noted above, most clinical studies or analyses have evaluated only short-term effect of diuretic use on renal function. Earlier large studies have linked long-term use of thiazide, loop diuretics, or their combinations to higher incidence of kidney failure requiring kidney replacement therapy or rapid decline in GFR (Table 3) [71–75]. Contrary to these results, in participants of the ALLHAT, neither calcium channel blocker nor ACEi was superior to chlorthalidone in reducing incidence of kidney failure or a composite of kidney failure with a 50% or greater decline in GFR [74]. In this post hoc analysis, participants assigned to receive amlodipine had a higher GFR than those assigned to receive chlorthalidone, although rates of kidney failure development were not significantly different among groups (Table 3). However, these previous data must be interpreted cautiously because most observational studies have been designed as single center studies with small sample sizes, short duration, and treatment selection biases. In addition, most randomized trials did not only target patients with CKD.

**Table 3:** Effects of diuretics on risk of kidney disease progression or kidney failure in subjects with CKD

Study	No. of subjects	Cohort	Follow-up	Renal function at baseline	Outcome measure	Results
Hawkins et al. [71] (2005)	Not reported	USRDS and IMS Health database	1980–2001	Not reported	ESRD incidence rate	Relationship between annual change in diuretic (predominantly, hydrochlorothiazide and furosemide) consumption and actual change in annual ESRD Incidence growth rate (r = − 0.754, P < 0.03)
Khan et al. [72] (2017)	621	A single center	2005–2014	eGFR 15–59 mL/min/1.73 m^2^	ESRD	More likely for CKD progression in diuretic (unspecified class) users (HR = 2.04, P = 0.01)
Khan et al. [73] (2017)	312	A single center	1 yr	eGFR ≤ 60 mL/min/1.73 m^2^	eGFR decline, progression of RRT	Larger annual eGFR decline in diuretic (loop diuretics in 48%, hydrochlorothiazide in 27%, furosemide plus hydrochlorothiazide in 25%) user Higher incidence of RRT in diuretic user
ALLHAT [74] (2005)	33,357	A randomized, double-blind trial	59.0 ± 16.5 mo	Mild reduction (60–89 mL/min/1.73 m^2^) and moderate-severe reduction (< 60 mL/min/1.73 m^2^) in GFR	ESRD incidence, GFR decrement of ≥ 50% from baseline	No differences in the incidence of ESRD or GFR decrement between chlorthalidone and amlodipine or lisinopril in reduced GFR groups
Fitzpatrick et al. [75] (2022)	47,666	Kaiser Permanente Northern California database	2008–2012	eGFR 15–59 mL/min/1.72 m^2^	ESRD, a composite renal outcome including reaching an eGFR < 15 mL/min/1.73 m^2^, 50% reduction in eGFR from baseline and/or ESRD	No significant association with durable reductions in eGFR in incident exposure to loop or thiazide diuretics in a diverse population with CKD compared with nondiuretic users

CKD, chronic kidney disease; USRDS, United States Renal Data System; ESRD, end-stage renal disease; eGFR, estimated glomerular filtration rate; HR, hazard ratio; RRT, renal replacement therapy; ALLHAT: Antihypertensive and Lipid-Lowering Treatment to Prevent Heart Attack Trial; GFR, glomerular filtration rate

In order to overcome such difficult challenges, a recent study has employed causal inference statistical methods to estimate the effect of using loop and thiazide diuretics on CKD progression, finally reporting no adverse effect of diuretic use in CKD patients [63]. Among 47,666 patients with eGFR 15 to 59 mL/min/1.73 m^2^ without previous receipt of loop or thiazide diuretics using database of Kaiser Permanente Northern California, neither initiation of diuretics nor type of diuretic was significantly associated with CKD progression or kidney failure after accounting for receipt of other medications and time-dependent confounders using marginal structural model with inverse probability weighting [75]. In real-world practice, patients with advanced CKD tend to be more prescribed diuretics in an effort to treat volume overload associated with CKD. Therefore, the higher rate of poor renal outcomes observed in CKD patients treated with diuretics might be attributed to the clinical situation where prescription of diuretics is nearly inevitable rather than diuretic use itself [75]. Possible explanations for different and conflicting findings according to the literature may include an increase of serum creatinine without a true reduction in GFR by diuretic-induced hemoconcentration, failure to fully account for all relevant covariates, treatment selection bias, age, and comorbidity profile [70, 75].

For the answer to question raised at case 2 presentation, an extensive literature review suggests maintaining existing diuretic therapy if prescribed doses of diuretic combination are currently appropriate for volume homeostasis and optimal blood pressure. Certainly, such decision should be individualized according to each patient profile considering age, comorbid conditions, concurrent medications, and potential long-term effects of diuretic exposure. Already advanced renal dysfunction at presentation as described in case 2, will ultimately precipitate the patient into a status of kidney failure requiring kidney replacement therapy in the near future. At least diuretic therapy might bridge CKD patients to dialysis or kidney transplantation. During that time, a process of shared decision-making with the patient about the type of kidney replacement therapy to use could be used [76].

## Conclusions

Considering that the overall prevalence of comorbid cardiovascular disease is high in patients with CKD [64], better blood pressure control by adding diuretics to existing antihypertensive regimens could reduce cardiovascular risk and further slow the progression of kidney disease [9]. While previous thought was that the use of diuretics was associated with poor renal outcomes independently of blood pressure, volume status, and other covariates [71–73], findings of a recent analysis are offering reassurance to patients with CKD receiving diuretic therapy [75].

Physicians tend to be always interested in developing first-in-class drugs and testing experimental medications. However, we should not lose our “old friends” or miss a chance to reconsider their forgotten talent. Although further long-term trials are needed to prove more favorable clinical outcomes with diuretics in patients with CKD, it becomes more evident that diuretics are useful for managing high blood pressure in both early and advanced CKD.

## Data Availability

Not applicable.

## Abbreviations

ACCOMPLISH: Avoiding Cardiovascular Events Through Combination Therapy in Patients Living with Systolic Hypertension
ACEi: Angiotensin-converting enzyme inhibitor
ALLHAT: Antihypertensive and Lipid-Lowering Treatment to Prevent Heart Attack Trial
ARB: Angiotensin-converting enzyme inhibitor
BPV: Blood pressure variability
CKD: Chronic kidney disease
CLICK: Chlorthalidone in Chronic Kidney Disease
DCP: Diuretic Comparison Project
eGFR: Estimated glomerular filtration
ESRD: End-stage renal disease
GFR: Glomerular filtration
HR: Hazard ratio
KDIGO: Kidney Disease Improving Global Outcomes
NCC: Na-Cl cotransporter
NESTOR: Natrilix SR Versus Enalapril Study in Type 2 Diabetic Hypertensives with Microalbuminuria
NKCC2: Na-K-2Cl cotransporter
RAS: Renin-angiotensin system
USRDS: United States Renal Data System
